# An Examination of the Performance of Blank Cartridges Used in Captive Bolt Devices for the Pre-Slaughter Stunning and Euthanasia of Animals

**DOI:** 10.3390/ani9080552

**Published:** 2019-08-14

**Authors:** Andrew Grist, Jeff A. Lines, Randall Bock, Toby G. Knowles, Stephen B. Wotton

**Affiliations:** 1 School of Veterinary Sciences, University of Bristol, Langford House, Langford, North Somerset BS40 5DU, UK; 2 Silsoe Livestock Systems, Wrest Park, Silsoe, Bedford MK45 4HR, UK; 3 Bock Industries, Inc. (BI), 156 Bock Lane, Philipsburg, PA 16866, USA

**Keywords:** animal welfare, blank cartridges, cartridge variation, captive bolt devices, mechanical stunning, performance, velocity measurement

## Abstract

**Simple Summary:**

In the United Kingdom, the main method of producing unconsciousness in cattle in abattoirs is the captive bolt device. This device comprises a piston (captive bolt) which is driven into the skull of the animal at a speed that renders it unconscious before it can perceive the impact. This speed of operation combined with unconsciousness allows for humane slaughter. The piston is driven forward either by compressed air or rapidly expanding gas from a blank cartridge, with the latter being the most common method. Blank cartridges produce gas by burning propellant and are available in a range of power (more propellant produces more gas which means more power). This paper examines variations in the performance of blank cartridges in producing sufficient velocity and therefore energy to stun animals, thereby affecting animal welfare at slaughter.

**Abstract:**

Blank cartridges provide the power source for the majority of captive bolt devices used for rendering animals unconscious prior to slaughter within the United Kingdom or euthanasia worldwide. This paper presents the results of the examination of cartridges as one of the variables that can contribute to an unsuccessful application of this method in practice. Variation was found in cartridge weight, propellant fill volume and velocity within boxes of 1000 cartridges. The variation found was greater in lower charge (1.00 grain) cartridges than in 3.00 grain cartridges, however velocity was found to be variable in both sets. For example, in vivo velocity measurements with 0.25″ calibre 3.00 grain cartridges demonstrated an average velocity of 50.8 m/s over 200 shots with a range of 35.7 to 62.9 m/s when used in the same device. This work demonstrates that variation in cartridge performance does occur and can be due to various factors such as fill volume and propellant function, and simply weighing cartridges cannot be used to determine function, therefore cartridge performance must be a factor that is considered in the event of a miss-stun.

## 1. Introduction

Mechanical stunning using a captive bolt device has been used extensively to produce immediate loss of consciousness in farmed livestock and has changed little in basic design since its first inception in the Behr Flash Killer of 1904 [1]. Cartridge powered captive bolt guns are used in abattoirs for all species, either as a first-choice method, or for back-up or, in an emergency. They are also used on-farm, as a stun/kill method for poultry [2,3] and for neonate pigs, goats and lamb [4,5,6]. The discharge of a blank cartridge provides a chemical energy source (gas pressure via exothermic deflagration of nitrocellulose in a confined space [7,8]) to a piston or bolt which imparts kinetic energy to the cranium of the animal, to induce an unconscious state through concussion [9,10]. Therefore, both penetrating and non-penetrating captive bolt devices produce a concussed state in the same manner, via impact, the only difference being that the subsequent penetration of the bolt in the former reduces the chance of recovery from the concussed state by mechanical damage to the brain [11].

It is important that any variability between shots is reduced to a minimum, to reduce the risk of a failure to concuss/stun, with its obvious welfare implications for the animal concerned. Variables that have been recognised as a cause of variation with use include: personnel training and competence, the level of maintenance of the captive bolt device, the correct choice of cartridge for the species and size, storage of the cartridges, head restraint and positioning of the device [12,13,14,15,16]. This paper is focused on the examination of the effect of the variability of one of these factors, i.e., the velocity developed by the cartridge used. Gregory et al. [9] reported that with the use of penetrating captive bolt for cattle, operatives are concerned about abnormally quiet discharge noise because they suspect that the shot will have been less effective. The authors suggest that one of the likely causes of a quiet discharge is insufficient nitrocellulose (propellant) in the cartridge. Gregory et al. [9] demonstrated that cartridges with less propellant produce less explosive noise and therefore quieter discharges, when compared with correctly filled cartridges. They reported a skew in the frequency distribution for the explosive noise measurements (dB) which indicated that about 4% of cartridges might be insufficiently filled. Gibson et al. [15] suggested that variation in cartridge fill may account for mis-stuns, especially with the lower grain cartridges. During research trials using lower grain cartridges (1.00 and 1.25 grain) for the euthanasia of neonate piglets, lamb and kids [3,4,5], variation in power was noted, which resulted in further investigation. This paper reports the findings of research to further investigate the variability present in commercially available cartridges, and the effect that variability may have on effective stunning.

### 1.1. Cartridge Description

**Figure 1:**
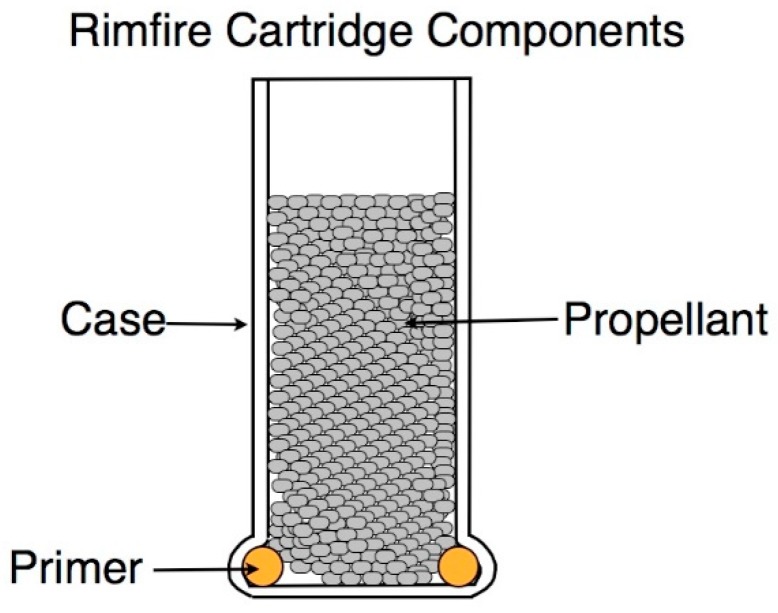
Rimfire cartridge components. Cross sectional diagram of an uncrimped blank cartridge.

A typical rimfire blank cartridge consists of three, separate components: the case, the primer and the propellant and no projectile. (Figure 1)

### 1.2. Case

The case is usually formed from sheets of cartridge brass (copper: zinc ratio of 70:30), crimped at the end to contain the primer and propellant [17]. The crimp is colour coded with lacquer to denote the nominal charge within the cartridge, however, this is not a hermetic seal. The calibre of the cartridge is the diameter of the case, usually quoted in inches (hence 0.22″ has a nominal diameter of 5.59 mm). To facilitate ignition of the primer compound by impact from the firing pin, the case is thinner at the rim; this designed weakness restricts rim-fire cartridges to use only for low pressure applications such as blanks used in captive bolt guns and starting pistols [8].

### 1.3. Primer

The primer consists usually of diazole and barium nitrate and is a small quantity of an impact- and friction-sensitive high explosive (not propellant) incorporated into a bead around the base rim of the cartridge by centrifugal force. The primer is ignited by striking the rim of the cartridge, hence the nomenclature of ‘rim-fire cartridge’ [8]. This explosive must generate both sufficient heat and a sufficiently powerful flame to ignite the propellant which has an autoignition temperature of 170–190 °C. Voids in the primer in the rim cavity can produce misfire.

### 1.4. Propellant

**Figure 2:**
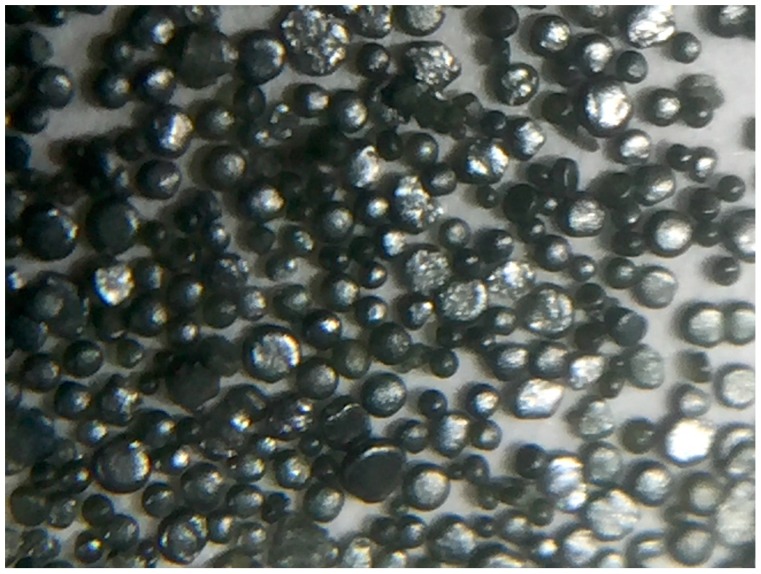
Propellant from a 0.22″ blank cartridge (10×).

The main propellant used in blank cartridges is 90–98% nitrocellulose, a smokeless single-base powder (as it contains one substance that produces energy) usually supplied as a fine flake powder. On combustion, smokeless powders are transformed almost entirely into gas: carbon dioxide (CO_2_), carbon monoxide (CO), water vapour (H_2_O), hydrogen (H_2_) and nitrogen (N_2_). Other chemical constituents are added to the nitrocellulose, such as graphite acting as a plasticizer and antistatic coating, potassium sulphate (K_2_SO_4_) added to reduce post combustion flash, diphenylamine (C_12_H_11_N) or ethyl centralite (C_17_H_20_N_2_O) as a stabilizer and dinitrotoluene (C_7_H_6_N_2_O_4_) which acts as a surface moderant to retard the initial burning rate, initial gas generation rate and the initial flame temperature [18,19]. A sample of propellant from a typical 0.22″ blank cartridge is shown in Figure 2.

**Figure 3:**
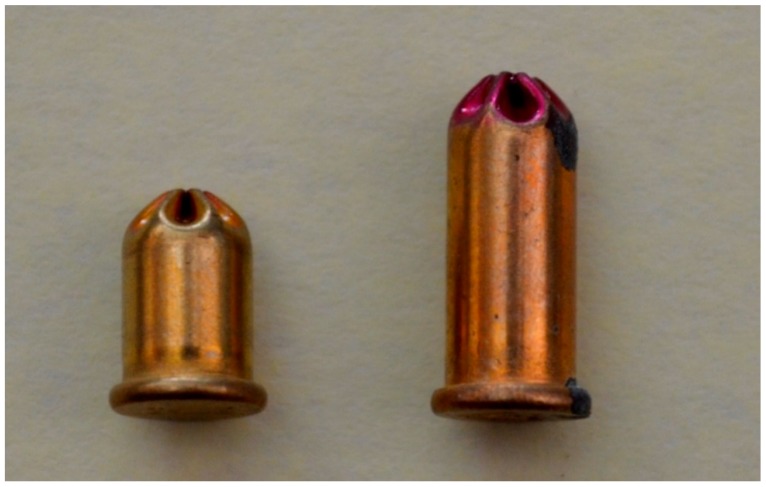
Temple Cox (manufacturer) 0.22″ blank cartridges: 1.00 grain cartridge on left; 2.00 grain cartridge on right.

Historically, the length of the case was altered to accommodate the propellant (Figure 3) but it is current practice for the same length case to be used for all cartridge strengths, with the void filled with a non-volatile filler such as silica [15]. Once ignited by the priming compound, the nitrocellulose deflagrates (propagation velocity below 100 m/s as opposed to detonation which occurs above 100 m/s). The propellant quantities contained in cartridges are quoted as the imperial measurement of grains (1.00 grain = 64.80 mg), with most manufacturers qualifying this measurement by quoting ‘nominal’ grain fill. Nitrocellulose will deteriorate over time in ambient conditions due to hydrolytic and/or thermal decomposition, an important factor when considering storage of cartridges with a non-hermetic seal in damp conditions. Although nitrocellulose itself is not water soluble, many of the additives, such as potassium sulphate are, and this can affect the burn rate.

### 1.5. Rationale

As one of the essential components of successful preslaughter stunning or euthanasia of animals, this paper examines commercially available cartridges to assess performance and uniformity of propellant fill weight within batches, to identify possible variation that could affect the cartridge performance and hence the ability to apply sufficient force to stun/kill every time. As part of the examination, variation in cartridge weight was assessed and then combined with velocity measurements to assess if cartridge weight could be a simple method of determining performance. This paper also discusses the possible causes of variation with cartridges. 

## 2. Materials and Methods 

This current investigation of cartridges comprises seven trials, using Accles and Shelvoke (Birmingham, UK) supplied 0.22″ and 0.25″ calibre blank cartridges for captive bolt devices taken from a batch (box of cartridges) to replicate standard use. The cartridges were within expiry dates and stored in dry conditions: Trial One—Cartridge weight, Trial Two—Cartridge weight before and after firing, Trial Three—Velocity measurement with a velocimeter, Trial Four—Velocity measurement (free flight projectile method), Trial Five—in vivo velocity measurement, Trial Six—Cartridge propellant fill volume assessment and Trial Seven—Cartridge case assessment post firing. All trial data were recorded in Microsoft Excel (Version 16.5 Microsoft Corporation, Redmond, WA, USA) for later analysis.

### 2.1. Trial One—Cartridge Weight

As part of the research project for the use of a non-penetrating captive bolt for the euthanasia of neonate piglets, lambs and kids, [4,5,6] four hundred cartridges were removed, taken from a box of 1000 Brown, 1.00 grain 0.22″ calibre cartridges and were individually weighed on a laboratory balance (Sartorius 1702 MP 8–1 Analytical Balance, Sartorius Stedim Systems GMBH, Guxhagen, Germany) with a stated precision of 0.1 mg.

### 2.2. Trial Two—Cartridge Weight before and after Firing

One hundred cartridges were removed from a box of 1000 green, 3.00 grain 0.22″ calibre cartridges and individually numbered and weighed twice on a laboratory balance (Sartorius ENTRIS124-1S Analytical Balance, 120 × 0.0001 g, Sartorius Stedim Biotech North America Inc., New York, NY, USA), and an average of the two weights was recorded. Each cartridge was fired (Trial Three) and reweighed; the cartridge case was subsequently cleaned with acetone and a swab and reweighed to give a measure of the residues left in the cartridge case after firing. Ninety-seven results were recorded, three results being discarded due to lack of a matching velocity measurement from Trial Three. These cartridges were selected as representative of commonly used cartridge size as the manufacturer quotes that they are suitable for medium sized animals.

### 2.3. Trial Three—Velocity Measurement (Velocimeter Method)

**Figure 4:**
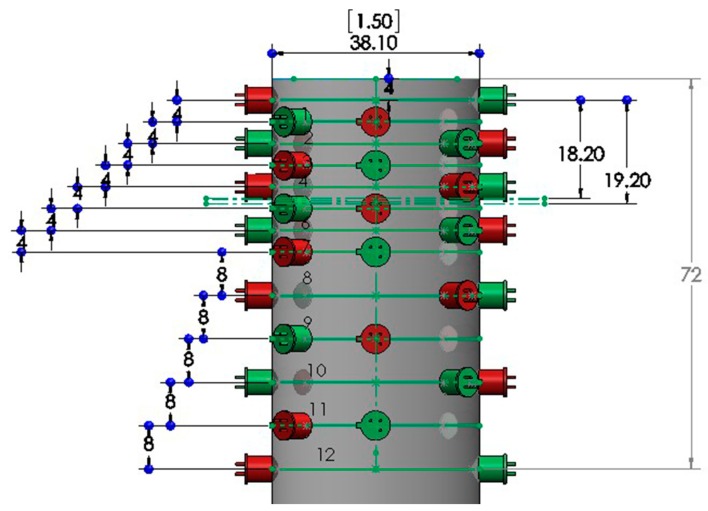
Diagrammatic representation of the bench velocimeter. Paired LED sensors, with green being the emitter and red being the receiver for each section. The gun to be tested is placed on the top of the velocimeter and the bolt turns on and off each LED sensor pair as it passes; the velocity of the bolt is then calculated.

**Figure 5:**
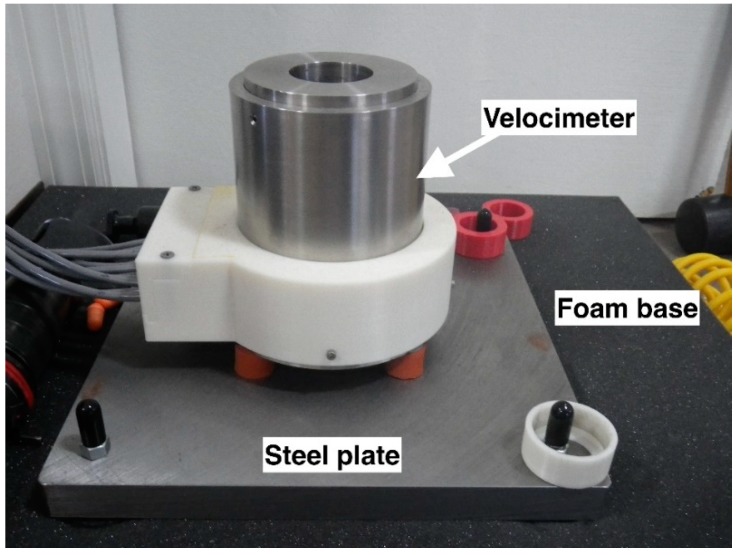
Velocimeter apparatus setup for firing.

A benchtop velocimeter was developed by Bock Industries (Philipsburg, PA, USA) to provide 12 discrete velocity points over the full travel of the penetrating bolt: with a velocity data point every 4 mm for the first 7 zones and then every 8 mm for the next 5 zones (Figure 4). This velocimeter was encased in a stainless-steel housing bolted to a steel plate attached to a foam base (Figure 5). The arrangement of a paired LED emitter and sensor provided an accurate measure of velocity as the bolt passed between pairs throughout the travel after firing.

Initially, ten 4.00 grain, three 3.50 grain and three 3.00 grain 0.25″ calibre cartridges were fired in an Accles and Shelvoke “Bulldozer” contact firing penetrating captive bolt device (Accles and Shelvoke, Birmingham, UK) and the velocities were measured and recorded.

One hundred 3.00 grain 0.22″ calibre cartridges were subsequently taken from a box of 1000 cartridges, weighed on a laboratory balance (Sartorius ENTRIS124-1S Analytical Balance, 120 × 0.0001 g, Sartorius Stedim Biotech North America Inc., New York, NY, USA). The cartridges were individually shot using an Accles and Shelvoke 0.22″ calibre Cash Special and the resultant velocities were measured and recorded using the benchtop velocimeter.

### 2.4. Trial Four—Velocity Measurement (Free Projectile Method)

In this trial three, outwardly identical 0.22″ CASH Small Animal Tools (Accles and Shelvoke, Birmingham, UK) were used. The non-penetrating head of the bolt was replaced with a cup and a spherical projectile. The bolts and knocker heads of the three guns tested had masses ranging from 177 to 179 g. These bolts under test, with the knocker head replaced by a cup and projectile, deviated from the mass of the bolt under operational conditions by less than 1%. The guns were oriented vertically for testing so that, before firing, the projectile sat in the cup and during the acceleration stage of the bolt, the projectile was accelerated with the bolt. As the bolt began to decelerate, due to the action of the recuperating sleeves, the projectile continued at a constant velocity in free flight over the measured distance. Its velocity was subsequently measured based on the time taken to pass through a pair of infrared beams at distances of 40 mm and 60 mm from the muzzle. The kinetic energy was calculated (E_K_ = ½ mass x bolt velocity^2^).

### 2.5. Trial Five—In Vivo Velocity Measurement

Two hundred bovine animals were shot using a 0.25” calibre bulldozer with green (4.50 grain) cartridges within a commercial abattoir. The baseplate of the Accles and Shelvoke “Bulldozer” penetrating captive bolt device was replaced with a prototype velocity recording device produced by Bock Industries (Philipsburg, PA, USA) as part of an ongoing research trial with the University of Bristol. Details of the velocity recording device are not produced in this paper as they are commercially sensitive.

### 2.6. Trial Six—Cartridge Fill Volume Assessment

Gregory et al. [9] and Gibson et al. [15] suggested there may be a variation in cartridge fill and the latter stated that silica is used to increase the fill volume of lower strength cartridges. Therefore, ten 4.00 grain and ten 1.00 grain cartridges were taken from boxes of 50 cartridges and the crimp carefully opened by hand. The propellant fill was separately emptied onto filter paper to visually compare the difference in fill volume.

### 2.7. Trial Seven—Case Deformity

**Figure 6:**
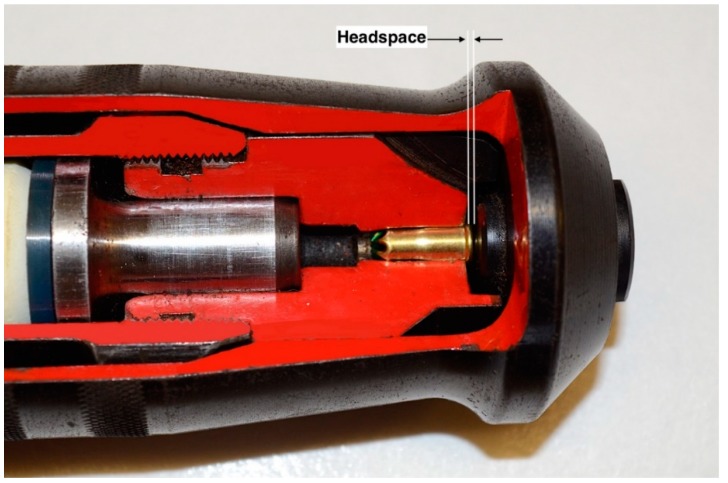
The headspace is the distance between the cartridge and the firing pin block (cross sectional model of an Accles and Shelvoke ‘Cowpuncher’ 5413R 0.22″ calibre contact firing penetrating captive bolt device).

Following the previous trials, it was noted that the cases of the spent cartridges were occasionally deformed. Deformation of the case on firing is due to excessive headspace in the device. When a rimfire cartridge is placed within the breech, the rim prevents the cartridge from fully entering the breech. The headspace is the distance between the breech and the firing pin (Figure 6). On firing, the cartridge is exposed to a rearward force equal to the pressure wave emanating from the front of the cartridge. This force will ‘seal’ the cartridge within the chamber to allow the expanding gas to propel the bolt forward. If there is excessive headspace, the cartridge is allowed to travel backward in the device and can deform. This rearward movement also increases the expansion chamber volume which will in addition, affect velocity, as was found in military weapons [20]. Random cartridge cases were examined after firing in Trial Three for deformation that could indicate rearward movement.

### 2.8. Data Analysis

Results are reported using simple descriptive statistics and graphically, where appropriate, to show frequency distribution.

## 3. Results

### 3.1. Trial One—Cartridge Weight (1-Grain—Brown Cap 0.22″ Calibre)

**Figure 7:**
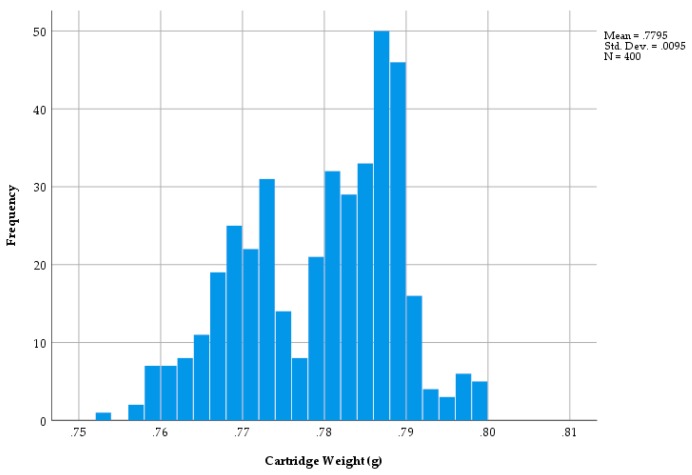
The variation in weight across a sample of 400 brown cap, 1.00 grain cartridges.

The distribution of the 1.00 grain cartridge weights followed a bimodal normal distribution (Figure 7). 

### 3.2. Trial Two—Cartridge Weight (3.00 Grain—Green Cap 0.22″ Calibre)

**Figure 8:**
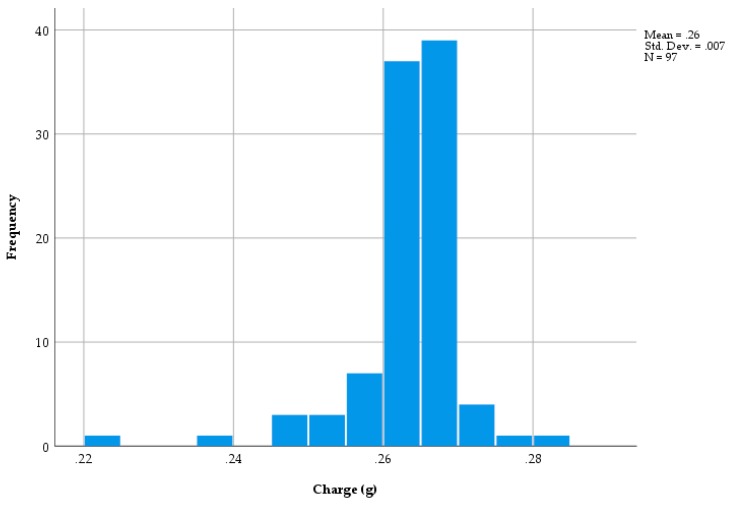
The distribution of charge weights (97 Green 3.00 grain cartridges) (g).

**Figure 9:**
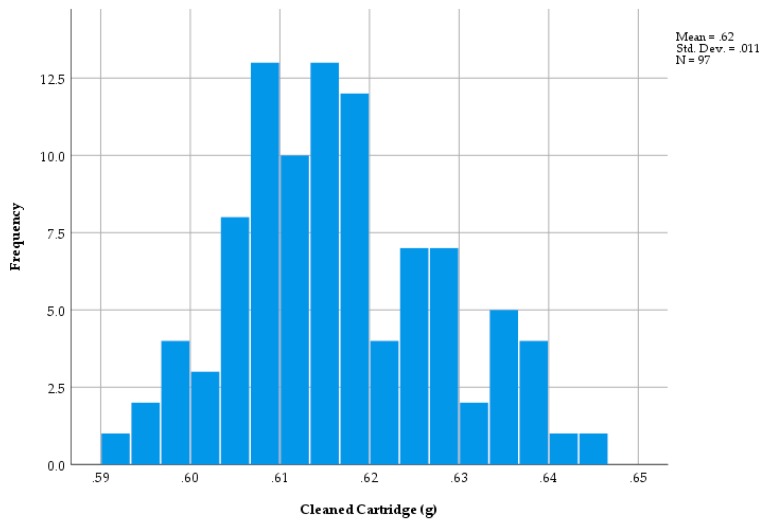
The weight distribution of the cleaned cases (g).

The cleaned post-firing cartridge weight was subtracted from the full cartridge weight of 97, 3.00 grain cartridges to give the weight of the charge. A histogram of charge weights is shown in Figure 8 which shows two outlying, low values of below 0.24 g with the remaining weights relatively tightly clustered about the mean. The distribution of the weights of the cleaned cartridges is shown in Figure 9, which shows a relatively normal distribution with a range in weights from 0.590 to 0.646.

### 3.3. Trial Three—Velocity Measurement (Velocimeter Method) 

**Figure 10:**
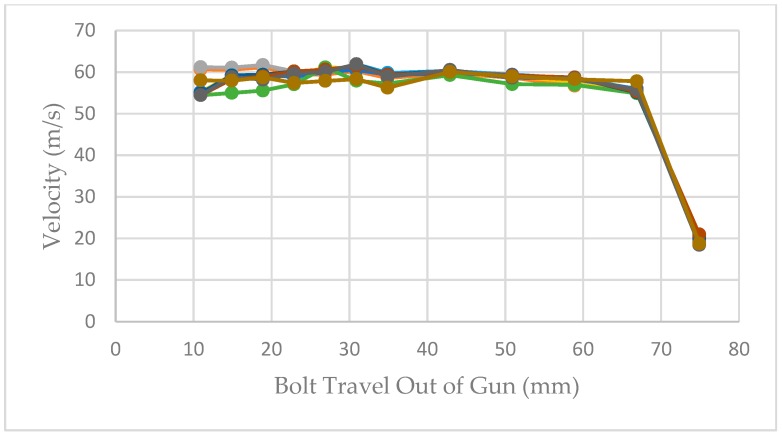
Bolt velocities recorded by the velocimeter at the different measurement points; 10 shots using 4.00 grain cartridges.

**Figure 11:**
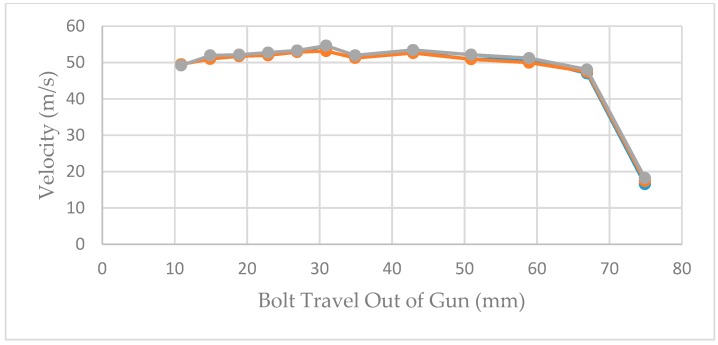
Bolt velocities recorded by the velocimeter at the different measurement points; 3 shots using 3.50 grain cartridges.

**Figure 12:**
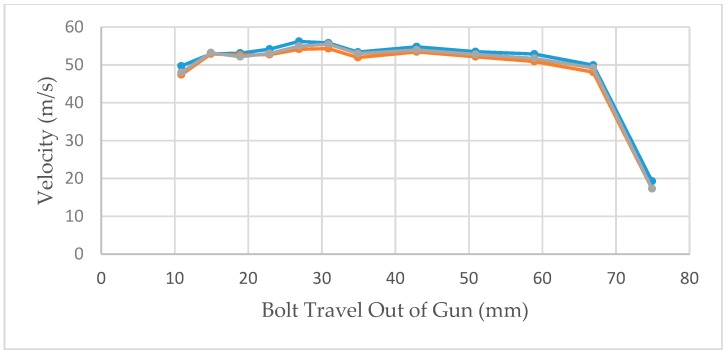
Bolt velocities recorded by the velocimeter at the different measurement points; 3 shots using 3.00 grain cartridges.

The velocities recorded at each measurement position in the initial trial using a 0.25″ calibre Cowpuncher with ten 4.00 grain, three 3.50 grain and three 3.00 grain 0.25″ calibre cartridges are shown in the three figures below. Figure 10, Figure 11 and Figure 12 show the velocities recorded for each of 10 × 4.00 grain cartridges, 10 × 3.50 grain cartridges and 10 × 3.00 grain cartridges, respectively, at each of the data points in the benchtop velocimeter. 

**Figure 13:**
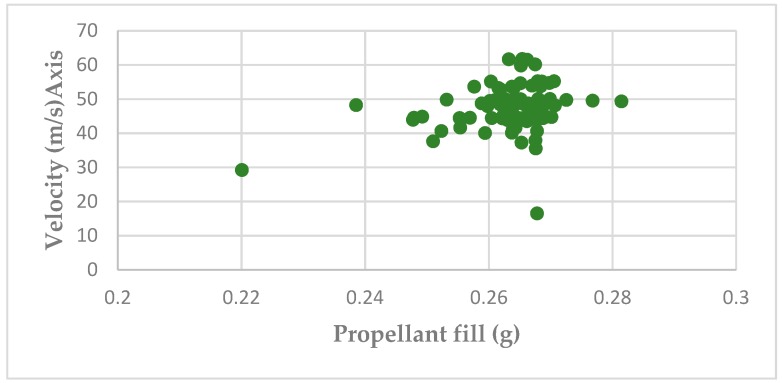
A scattergram of the relationship between propellant fill (g) and velocity (m/s) using 100 Green 3.00 grain cartridges.

Figure 13 shows the scattergram of bolt velocity and propellant fill generated by a random selection of 100 Green 3.00 grain cartridges together with the individual pre-firing cartridge weights (g). Analysis of the data showed that there was a significant (*p* < 0.005) positive linear regression between the cartridge weights and the velocity (Velocity (m/s) = −16.546 + (Grain × 15.764), however, the relationship was weak with an adjusted R square of only 0.08. For the analysis, the extreme grain value of <3.5 and the extreme velocity of <20 m/s were excluded. 

### 3.4. Trial Four—Velocity Measurement Free Flight Method

**Figure 14:**
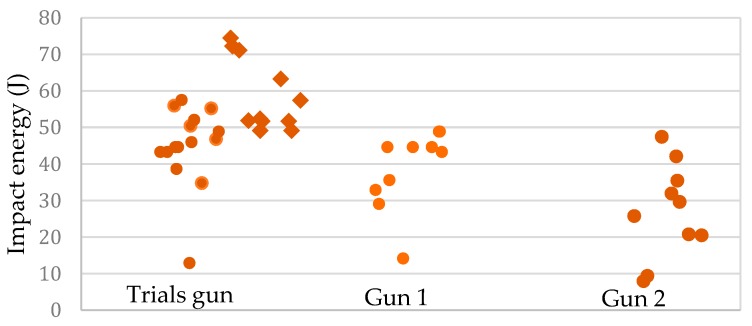
Impact energy for three Accles and Shelvoke Small Animal tools using brown 1.00 grain cartridges. Trial gun energies shown with recuperating sleeves cold (round markers) and warm (diamond markers).

Figure 14 shows the bolt impact energy levels measured for the three guns (Accles and Shelvoke Small Animal Tool) calculated from the maximum velocity achieved by the bolts. The measurements of the Trials gun were made with the gun cold (round markers in Figure 14) and also after an extended period of use when the gun felt warm in the hand (diamond markers in Figure 14). Figure 14 shows the kinetic energies recorded with 1.00 grain cartridges. The mean energy values for 1.00 grain cartridges with a full set of cold recuperating sleeves were as follows:

Study Gun 47 ± 6 J
Gun 1    41 ± 7 J
Gun 2    32 ± 10 J

The values above exclude all measurements of kinetic energy delivering less than 15 Joules. These data indicate several sources of variation in kinetic energy. Under nominally identical conditions, a significant spread in the energies is observable. This is likely to be due either to variations in the cartridge fill, or to variations in the way the kinetic energy is converted in the gun from shot to shot.

It appeared that a significant proportion of the cartridges (4 out of 41) were faulty (Figure 14), delivering only 4 to 14 J of kinetic energy. While these cartridges did fire, they did not seem to have contained the correct quantity of functioning charge. 

### 3.5. Trial Five—In Vivo Velocity Measurement

**Figure 15:**
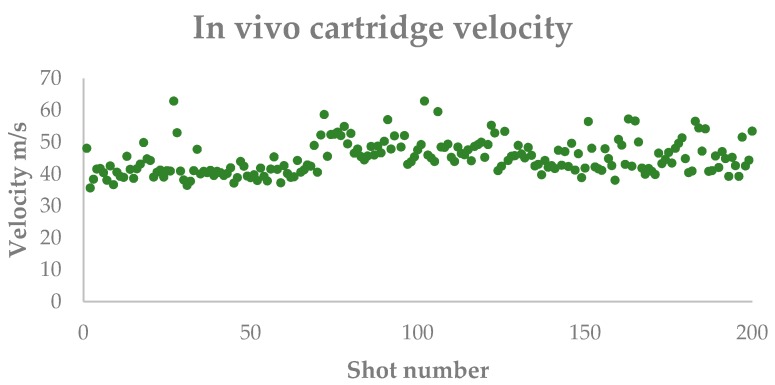
In vivo velocity measurements for 200 cattle shot with green 4.50 grain cartridges in an Accles and Shevoke ‘Cowpuncher’ penetrating captive bolt device fitted with the experimental velocimeter.

Over the two hundred ‘real life’ applications measured at the abattoir, the bolt velocity ranged from 35.7 to 62.9 m/s with an average velocity of 50.8 m/s (Figure 15).

### 3.6. Trial Six—Cartridge Fill Volume

**Figure 16:**
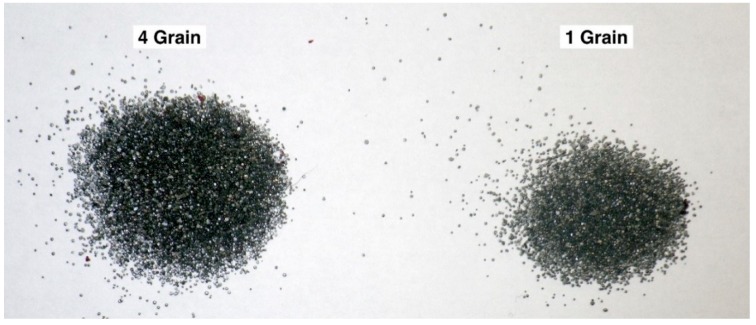
Cartridge contents comparison between a 4.00 grain and a 1.00 grain 0.22” calibre cartridge. Both cartridge cases are the same length and volume.

An example of the cartridge content from a 4.00 and 1.00 grain cartridge is shown in Figure 16, demonstrating the variance in cartridge propellant fill volume encountered between grain loads and that there appeared to be a lack of ‘filler’ material in the 1 grain cartridges.

### 3.7. Trial Seven—Case Deformity Due to Excessive Headspace

**Figure 17:**
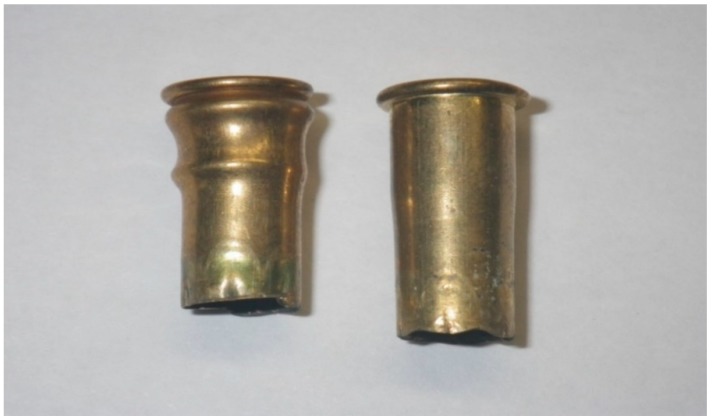
Deviation of cases fired in the same device. Right hand case displaying rim end expansion as expected to seal the case within the breech; left hand case showing deformity and signs of having moved backward on firing.

**Figure 18:**
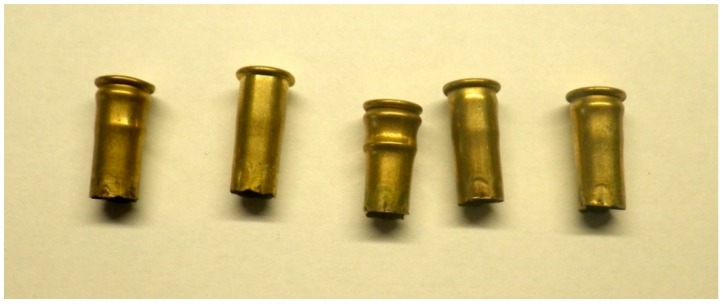
Range of deformities seen in cartridge cases fired using the same device.

Case deformity was encountered in cartridges post firing from the same device (Figure 17 and Figure 18). The deformities encountered were not correlated in this study to specific firings, as the deformity was noted after Trial 3 had taken place.

## 4. Discussions

In *Trial One Cartridge Weight (1.00 grain cartridges),* the results demonstrated a bimodal distribution of cartridge weights, which is not the expected results for a product that is meant to be uniform. However, there was an apparent drop in the frequency of cartridges produced at a weight of approximately 0.7750 g with a total range of approximately ±25 mg about this weight. The implications of this distribution with regards effectiveness are unknown; a discussion with the manufacturer would be required to understand how this bimodal pattern had arisen. 

### 4.1. Trial Two—Cartridge Weight before and after Firing (3.00 Grain Cartridges) 

It was postulated that the range in cartridge weights could be produced predominantly by variation in casing weights, however, the overall range in casing weights of 50 mg (0.593 to 0.643 g) shown in Figure 9 and in charge of 61 mg (0.220 to 0.281 g) shows that the differences recorded were due, in approximately equal parts, to the variations in both components. As a percentage of mean weight, the variation in charge was considerably larger for fill (±11.6%) than for the case (±4.0%), however, given the equal contribution to overall weight, simply weighing the cartridge did not give an indication of fill.

### 4.2. Trial Three—Velocity Measurement (Velocimeter Method)

The effect of velocity measurement at different measurement points of the bolt extension shown in Figure 10, Figure 11 and Figure 12 follows very similar patterns with the different cartridges tested. The velocity appears to be fairly consistent between measurement points 11–59 mm of the bolt extension but as expected fall away as the effect of buffer compression slows the bolt before returning it into the breech.

The variation in cartridge weight expressed in grammes in Figure 13 and the resultant bolt velocity shows additional cause for concern. This is because the data indicate that low velocity and hence low energy impact can result from either a cartridge of normal weight or, to one of reduced weight. This would suggest that simply weighing the blank cartridges would not identify all the cartridges that would produce reduced velocity/energy upon firing.

### 4.3. Trial Four—(1 Grain Cartridges) Free Flight Method

It appears that a significant proportion of the cartridges (4 out of 41) were faulty (Figure 14), delivering only 4 to 14 J of energy. While these cartridges did fire, they seem not to have contained the correct quantity of propellant. These data provide no evidence to suggest that the warming of the buffers is responsible for the bulk of the inter-shot variation. Further investigation would be needed to clarify the situation. 

The results from this trial correspond to those of Gibson et al. [15] that demonstrated a wide variation in performance of lower powered cartridges, with 9.76% of the cartridges delivering 4–14 J compared to an expected output of 76 J ± 15% according to the latest data sheet (Frontmatec Accles and Shelvoke). 

### 4.4. Trial Five—In Vivo Velocity Measurement

Daly et al. [11] suggested that 55 m/s should be considered as the minimum velocity to stun cattle with an Accles and Shelvoke Ltd. captive bolt device using Visual Evoked Responses as a conservative indicator of brain dysfunction. As the animal is concussed due to the impact transfer of kinetic energy (E_K_) to the cranial vault and E_K_ = ½ mass × bolt velocity^2^, the variance in velocity will have a greater impact on the ability to stun than the mass of the bolt. The average bolt velocity measured in vivo during the 200 applications within an abattoir was 50.8 m/s with a peak of 62.9 m/s, however the lower velocities measured (35.7 m/s minimum) fall below this level; in effect, the lower velocity shot will have three times less energy than the fastest within this batch. The animals were assessed by two experienced researchers (Grist and Wotton) for the effectiveness of the stun using the standard behavioural indicators of loss of posture, no corneal reflex, no pain response and no rhythmic breathing [10,16] and the results corresponded to the findings of von Holleben et al. [21] that the failure to observe the minimum recommended velocity did not correspond to a failure to stun. However, the trial provided evidence of a wide variation in velocity in vivo of the same size cartridges from the same batch fired in the same device. We believe that that the development of the in vivo velocimeter will enable abattoir operatives to be informed of the effectiveness of each shot and for that data to be recorded to give a long-term evaluation of the performance of the gun and monitoring of cartridge batches once the device is in use and velocities can be correlated to stun failure. The results from this development will lead to the production of a practical system that will be made available to the meat industry to either retro-fit to existing guns or, to be incorporated into the design of new models. It is anticipated that the results will be published in an appropriate scientific journal. The benefit to the industry will be to advance animal welfare monitoring during mechanical stunning of all species. This device would enable far greater control over the process and permit Animal Welfare Officers (AWO) to closely monitor the performance of captive bolt guns and cartridge batches and initiate maintenance and/or, replacement before the gun fails. The data produced will also meet current legislative requirements in Europe [22,23,24,25].

### 4.5. Trial Six—Issues Arising from Cartridge Fill Volume 

The fact that the lower strength cartridges contain a lower volume of propellant fill than high grain cartridges is likely to vary the performance of the former. As previously discussed, the case size is standard across the different strength cartridges and with lower strength cartridges, the case is not completely filled which affects the loading density (the ratio of the weight of powder charge to the capacity of the case). Issues of low loading density include erratic ignition, change in the pressure curve (moving the peak towards the muzzle), or even overly rapid burning (“detonation”) of the powder charge. As with all blank-fired captive bolt devices, the gun is applied with the muzzle pointing down for operation; the lower strength cartridges will allow the propellant to move to the crimped end of the case. This means that there is a greater distance between the priming compound and the propellant, which will affect the ignition and burn rate of the propellant and may lead to propellant burning within the expansion chamber behind the bolt as the latter moves forward. Although all the propellant may burn, it will not burn fast enough to provide the required initial pressure to the captive bolt, resulting in a lower bolt velocity and may affect the ability of the bolt to deliver sufficient kinetic energy to the cranium to stun. Loading density and the effects of reducing this are well known in the shooting fraternity, with self-loaders in competitive shooting being aware that this can affect accuracy by altering the ballistics of the propellant burn [20].

### 4.6. Trial Seven—Case Deformity

Anecdotally, and having been witnessed by the authors in several abattoirs, there is a practice, with contact firing varieties of captive bolt devices, of using the same barrel cap for both breeches that have to be in position at the stunning point; the back-up device being a preloaded breech which has the same barrel cap fitted for operation. Although barrel caps are not matched to specific barrels, wear of the barrel cap with use may produce a looser fit. Cartridges should be examined after firing for evidence of case deformity due to excessive headspace and the barrel cap corresponding to the breech should be used for secondary stuns. The further development of the in vivo velocimeter will enable more research to be undertaken to establish if there is a link between lower shot velocity and case deformation due to headspace.

### 4.7. Case Splitting

**Figure 19:**
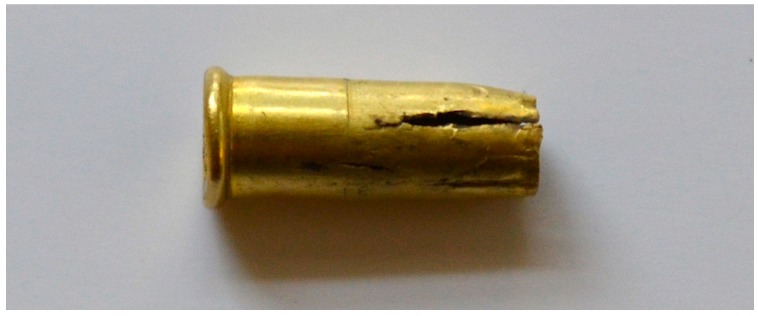
Case splitting in an AS head stamped 0.22″ calibre cartridge.

**Figure 20:**
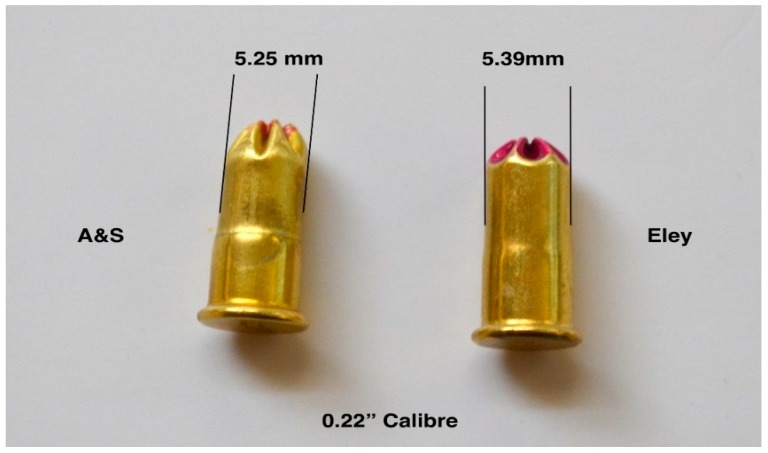
Variation in case diameter between AS and Eley cartridges for use in the same 0.22″ calibre device.

**Figure 21:**
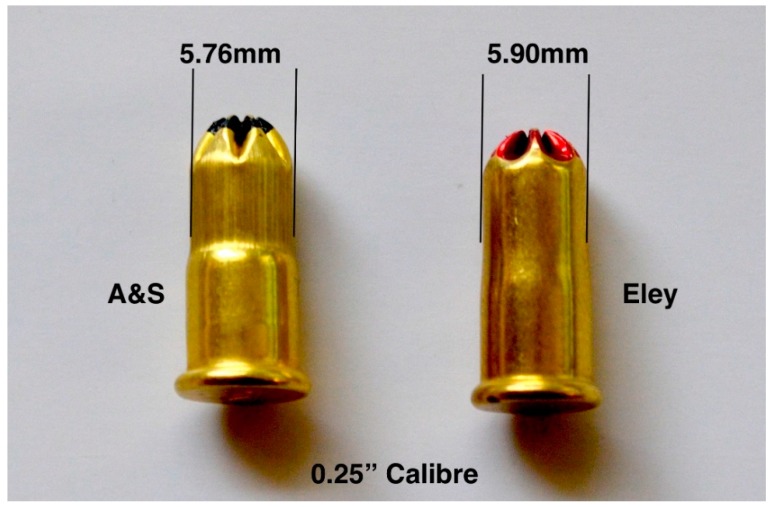
Variation in case diameter between AS and Eley cartridges for use in the same 0.25″ calibre device.

Recent anecdotal reports from some abattoirs suggest that blank cartridges were occasionally sticking in the breech after firing. It was proposed that this was likely caused by cartridges splitting along their length (Figure 19). The cartridge heads that were stamped E (Eley) did not split, but some of those head stamped AS (Accles and Shelvoke) were found to have split upon removal from the breech. Upon examination, it was found that the new AS cartridges had a more pronounced shoulder that reduced the diameter of the case front by 0.14 mm in both the 0.22″ and 0.25″ calibre cartridges when compared to the corresponding Eley cartridges (Figure 20 and Figure 21). In addition, we noted some evidence of stress lines due to the forming process. These cartridges were not tested in velocity trials. 

### 4.8. Variation in Cartridge Performance

This research found that there is a variability in cartridge performance across all ranges, with lower strength cartridges demonstrating more variability in bolt velocity than high grain cartridges. The mean cartridge weight (0.7795 g—Figure 7) represents the standard weight when a ‘nominal’ target charge of 0.065 g (1.00 grain) of nitrocellulose. The range of 0.7999–0.7537 g (Figure 7) produced a range in weight of 0.0462 g and if the mean cartridge weight (0.7795 g) equates to a fill of 0.065 g nitrocellulose, then 0.7795 − 0.7537 = 0.0258 which would represent 0.0258/0.065 × 100 = 40% reduction in fill. 

Given that it is current practice for the same length case to be used for all cartridge strengths and that the variability in the cleaned cartridge case weight was measured as 0.590–0.646 g (Figure 9) for 3.0 grain cartridges, i.e., ±4.5%, this would suggest that the majority of the weight differences were due to differences in the weight of the propellant.

The mean measured charge weight of 0.26 g (range 0.220–0.285—Figure 9) for a 3.00 grain cartridge shows two low outliers, which may explain the variation in velocity shown in Figure 13. This mean measured charge weight (0.26 g) for a 3.00 grain cartridge can be compared to a ‘nominal’ cartridge fill of 0.195 g (3 × 0.065 g). This difference is difficult to explain.

It is suggested that the result of firing a captive bolt gun with a cartridge that has potentially 40% of the required nitrocellulose may not be audibly detected by the stunning operative but is highly likely to result in an ineffective stun [9]. Abattoirs are required by retailers to record the incidence of double-shots, therefore this occurrence is not only a welfare issue but can also result in punitive action against the slaughter-person.

Physical examination of the cartridges found variability in fill weight, fill volume and case deformity on firing. The question is, is that variability important in the context of the job the cartridges are supposed to be doing? The study of grain against velocity showed that loading does have some weak influence on velocity, however, an R square of 0.08 means it only explains 8 per cent of the variability. Therefore, there are other factors driving the bulk of the variability in velocity. That given, when a cartridge with unusually low grain was used (as seen in Figure 14), even though it is infrequent, it does result in an unacceptably low velocity. 

In a situation where the protection of animal welfare by ensuring a stun occurs first time is a paramount concern, the uniform and reliable function of the blank cartridge is an integral part of the process but has been partially overlooked. The future development of the real-time in vivo velocimeter will allow more in-depth investigation into the variables encountered during this investigation, including the role that case deformity due to headspace and this rearward movement of the cartridge may have on bolt velocity and therefore kinetic energy delivered to the animal, the effect of age of cartridge and storage conditions. The in vivo velocimeter will also allow data to be gathered to assess the lower borderline velocity at which the device does not stun, which will allow warnings to be given to the operative in addition to the behavioural indicators they use to assess the stunned state [20]. 

## 5. Conclusions

The cartridge is a component and vital part of the stunning process; UK and EU legislation [22,23,24,25] emphasise the importance of cartridge strength as a primary parameter for a successful stun and for protecting animal welfare. This investigation, using new cartridges, demonstrated a variability in performance which will only be exacerbated by the environmental and storage conditions within the abattoir setting, including moisture, cartridge age and ambient temperature (as the temperature of the propellant before ignition can have an effect on burn rate) [20]. In Annex 1 of EC1099/2009 on the protection of animals at the time of killing, bolt velocity is listed as a key parameter for penetrative and non-penetrative captive bolt devices [22]. Currently, there is no method of measuring and recording this parameter in vivo. There is available bench testing equipment to measure velocity, such as the Stuncheck (Accles and Shelvoke, Birmingham, UK), but this does not provide information for every shot. It is therefore recommended that the further development of a method of recording every shot in vivo, within a commercial setting, should be encouraged to allow real time recording of velocity.

## 6. Patents

The Bock Industries Velocimeter will be patented, and as such the setup and methodology is not described in detail in this paper.
